# Tankyrases/β-catenin Signaling Pathway as an Anti-proliferation and Anti-metastatic Target in Hepatocarcinoma Cell Lines

**DOI:** 10.7150/jca.30976

**Published:** 2020-01-01

**Authors:** Jianghai Huang, Qiang Qu, Yong Guo, Yuqi Xiang, Deyun Feng

**Affiliations:** 1 Department of Pathology, the Second Xiangya Hospital.; 2 Department of Pathology, School of Basic Medical Sciences.; 3 Department of Pharmacy, Xiangya Hospital.; 4 Department of neurosurgery, Xiangya Hospital.; 5 Department of Pathology, Xiangya Hospital, Central South University, Changsha city, Hunan province, China.

**Keywords:** Tankyrases, β-catenin, metastatic, invasion, EMT, HCC

## Abstract

**Objective:** The Wnt/β-catenin pathway is involved in the development of hepatocellular carcinoma (HCC) and malignant events such as the epithelial-mesenchymal transition (EMT), metastasis, and invasion. Studies have illustrated that the inhibition of tankyrases (TNKS) antagonizes Wnt/β-catenin signaling in many cancer cells.

**Methods:** The expression levels of proteins related to the Wnt/β-catenin pathway and EMT were analyzed by immunohistochemistry in HCC tissue and paired adjacent normal tissue (n = 10), and in an analysis of The Cancer Genome Atlas (TCGA) data. Additionally, after treatment of HCC cell lines with TNKS1/2 small interfering RNA (siRNA) and a novel TNKS inhibitor (NVP-TNKS656), cell viability, cell clone formation, wound-healing, and cell invasion assays were performed.

**Results:** Higher expression of β-catenin, TNKS, vimentin, and N-cadherin was observed in HCC tissue compared to adjacent normal tissue, but lower expression of E-cadherin was found in HCC tissue. These findings were also observed in the TCGA analysis. In addition, TNKS inhibition (using TNKS1/2 siRNA and NVP-TNKS656) not only abrogated the proliferation of the HCC cell lines but also suppressed metastasis, invasion, and EMT phenotypic features. Moreover, the mechanisms related to TNKS inhibition in HCC probably involved the stabilization of AXIN levels and the downregulation of β-catenin, which mediates EMT marker expression.

**Conclusion:** The TNKS/β-catenin signaling pathway is a potential anti-proliferation and anti-metastatic target in HCC.

## Introduction

Hepatocellular carcinoma (HCC), which has poor prognosis and a high mortality rate, is one of the most common causes of cancer-related death in the world 1. The activation of the Wnt/β-catenin pathway has frequently been observed in HCC development 2, 3. The canonical Wnt/β-catenin signaling pathway, a well-known oncogenic pathway, is activated by stabilizing the transcriptional co-activator β-catenin (CTNNB1) by preventing its phosphorylation-dependent degradation 3. In a normal steady state, a multifactor β-catenin destruction complex is steadily assembled by several components, including β-catenin, the scaffold protein AXIN, the tumor suppressor adenomatous polyposis coli (APC), glycogen synthase kinase 3 beta (GSK3β), and casein kinase 1 alpha 1 (CSNK1A1) 3. Additionally, β-catenin interaction with the cell adhesion molecule E-cadherin at the cell-cell junction is involved in mechanisms regulating cell-cell adhesion, mobility, and proliferation 4, 5. Mutations or aberrant expression of the components of the β-catenin destruction complex cause HCC and increase epithelial-mesenchymal transition (EMT), distant metastasis, and invasion 6.

Two tankyrase (TNKS) isoforms,TNKS1 and TNKS2, belonging to a group of enzymes called poly ADP ribosyl polymerases (PARPs) 7 share overlapping functions and similar structures, including the ankyrin (ANK) repeat domain, the sterile alpha molecule (SAM) domain, and the catalytic PARP domain 8. In the Wnt/β-catenin pathway, TNKS PARsylates AXIN, which results in proteasome complex-mediated AXIN degradation after ubiquitination by the ubiquitin E3 ligase RNF146 9, 10. Many reports have shown that TNKS inhibition stabilizes AXIN and antagonizes Wnt/β-catenin signaling in various cancers, such as lung cancer 11, gastric cancer 12, 13, bladder cancer 14, astroglial brain tumor 15, pancreatic adenocarcinoma 16, breast cancer 17, bone cancer 18, and colon cancer 19, 20.

With the advent of novel inhibitors of TNKS, TNKS can act as a novel target in various cancers. The TNKS inhibitors XAV939 and WXL-8 attenuate WNT/β-catenin signaling and inhibit HCC cell growth 21, 22. NVP-TNKS656 was reported to be an orally active antagonist of TNKS and Wnt pathway activity in the mouse mammary tumor virus (MMTV)-Wnt1 mouse xenograft model 23. In the present study, we investigated the antitumor efficacy of TNKS small interfering RNA (siRNA) and NVP-TNKS656 in HCC cell lines, and we demonstrated that TNKS inhibition not only inhibited the proliferation of these cells but also suppressed their metastasis, invasion, and EMT phenotypic features.

## Materials and Methods

### Materials

TNKS, β-catenin, AXIN, vimentin, E-cadherin, and N-cadherin antibodies were purchased from Sigma-Aldrich and Abcam (Shanghai, China). NVP-TNKS656 was purchased from CSNpharm (#CSN13750, Shanghai, China).

### Cell line culture and HCC sample collection

The HCC cell lines SMMC-7721 and MHHC-97h were purchased from ATCC and cultured in Dulbecco's Modified Eagle Medium (DMEM; Hyclone) containing 10% heat-inactivated fetal bovine serum (FBS; Hyclone) and 2 mM L-glutamine (Gibco). Both cell lines were maintained in an incubator at 37°C in a fully humidified atmosphere of 5% CO_2_.

Ten HCC samples with adjacent tissue samples were obtained from 10 HCC patients at the Second Xiangya Hospital, Central South University. Informed consent was obtained and the study was approved by Ethics Committee of the Second Xiangya Hospital (no. 2019026-18).

### UALCAN web-portal gene expression and survival analyses using The Cancer Genome Atlas (TCGA) data

TNKS/β-catenin pathway-related genes and EMT-related genes (including β-catenin, TNKS1, TNKS2, vimentin, E-cadherin, and N-cadherin) were analyzed using the UALCAN web-portal (http://ualcan.path.uab.edu) and TCGA HCC subgroup data of individual stage. Heat maps of differentially expressed genes in HCC and adjacent normal tissues were created. Each gene expression level was represented as log_2_ (transcript count per million [TPM]+1). Box-whisker plots were used to show the gene expression in the HCC subgroup compared to adjacent normal tissues. Gene-level correlations with patient overall survival were also conducted. The TCGA HCC patient survival data were used for Kaplan-Meier survival analyses and for generating overall survival plots in the UALCAN web-portal.

### Immunohistochemistry (IHC) analyses

The expression of β-catenin, TNKS, vimentin, E-cadherin, and N-cadherin was detected in the 10 HCC samples and adjacent normal tissues by IHC, as described previously 21. A secondary horseradish peroxidase (HRP)-conjugated antibody (Maixin Reagent Co. Ltd., Fuzhou, China) and a 3,3-diaminobenzidine (DAB) staining kit (Maixin Reagent Co. Ltd.) were used in the IHC analyses.

### Molecular docking

The structure of the TNKS inhibitor NVP-TNKS656 (CID 136237316) and TNKS1 (Protein Data Bank [PDB] code 4LI6) were obtained from PUBCHEM (https://pubchem.ncbi.nlm.nih.gov/) and PDB (http://www.rcsb.org/), respectively. The docking analysis was conducted as described in the previous study 24.

### Cell viability

Each of the two HCC cell lines was seeded in 96-well plates, with 1000 cells in 200 μl DMEM per well. The cells were then treated with 0.5% dimethyl sulfoxide (DMSO) alone (used as the vehicle control) or various concentrations of NVP-TNKS656 (from 1.25 to 100 μM). After 3 days of incubation, the media and compounds were removed and replaced with 100 μl fresh DMEM and 20 μl Cell Counting Kit (CCK)-8 Reagent (Beyotime, Shanghai, China). After incubation for 2 h, the absorbance at 450 nm was measured using a microplate spectrophotometer.

### TNKS1/2 knockdown using siRNA

Pairs of independent double-stranded TNKS1 and TNKS2 siRNAs were identified in a previous study and synthesized by GenePharma Co. Ltd. (Suzhou, China). The siRNA sequences were as follows: TNKS1 sense strand 5'-GCAUGGAGCUUGUGUUAAUUU-3' and anti-sense strand 5'-AUUAACACAAGCUCCAUGCUU-3'; TNKS2 sense strand 5'-GAGGGUAUCUCAUUAGGUAUU-3' and anti-sense strand 5'-UACCUAAUGAGAUACCCUCUU-3'. To prepare the siTNKS1/2 mixture, the TNKS1 and TNKS2 siRNA were denatured at 95 °C for 5 min, annealed at room temperature for 30 min, and then mixed together to create the siTNKS1/2 mixture. Transient transfections of 50 nM siTNKS1/2 mixture and scrambled siRNA (siRNA negative control [siNC]) were accomplished using Lipofectamine 2000 reagent (#11668019, Thermo Fisher Scientific) according to the manufacturer's protocol.

### Cell clone formation assay

HCC cell lines treated with NVP-TNKS656 or transfected with the siTNKS1/2 mixture were reseeded in a new six-well culture plate at a density of 200 cells/well and incubated for 7-9 days. Fresh DMEM containing various concentrations of NVP-TNKS656 (0.125-20 μM) was added every 2 days. The six-well plate was then washed with phosphate-buffered saline (PBS) and colonies were stained with 0.5% crystal violet in PBS.

### Wound-healing assay

For the assessment of the migration ability of HCC cells, SMMC-7721 and MHHC-97h cells were seeded in six-well plates with DMEM containing 5% FBS and grown to 90% confluence. After the media were aspirated, a yellow pipette tip was used to make a straight scratch in each monolayer. Subsequently, the cellular debris was washed with PBS twice. The cells were exposed to NVP-TNKS656 or transfected with siTNKS1/2 for 24 h, respectively, as described above.

### Cell invasion assay

One hundred microliters of matrigel (0.4mg/ml) (Corning, cat no. 356234) was loaded into the upper chamber of a Transwell plate (Costar, USA, cat no. 3422) and incubated for 24 h. HCC cell lines were treated with NVP-TNKS656 or siTNKS, as described above, and then trypsinized to create a cell suspension. For the lower chamber, 750 µl DMEM with 10% FBS was added. Meanwhile, 200 µl cell suspension cells (1 × 10^5^ cells/ml) were added to the matrigel-coated upper chamber and incubated for 36 h. After aspirating the medium from the upper chamber and washing twice with PBS, the cells were permeabilized with 100% methanol for 20 min at room temperature. The methanol was then aspirated and the chambers were washed twice with PBS. Non-invaded cells were scraped off with cotton swabs. The invasive cells were stained with 0.5% crystal violet for 20 min. After the crystal violet was removed and the invasive cells were washed twice with PBS, the invasive cells in four random fields under a bright light microscope were counted.

### Western blotting

After treatment with NVP-TNKS656 or the siTNKS1/2 mixture, MHCC-97h cells were lysed using radioimmunoprecipitation assay (RIPA) buffer (Beyotime). Protein concentrations were assayed using bicinchoninic acid (BCA) protein assay reagent (Beyotime). The western blotting process was based on a previous report 25 , and it included sodium dodecyl sulfate polyacrylamide gel electrophoresis (SDS-PAGE), transfer onto polyvinylidene fluoride (PVDF) membranes, blocking, antibody incubation, and blotting band detection.

### Statistical analysis

SPSS 13.0 statistical software (SPSS, Inc., Chicago, IL, USA) was used for the statistical analyses. Quantitative variables were expressed as mean ± SD and analyzed with t tests. P-value <0.05 was considered statistically significant.

## Results

### TNKS/β-catenin pathway and EMT gene expression in HCC and normal tissues

**Figure 1:**
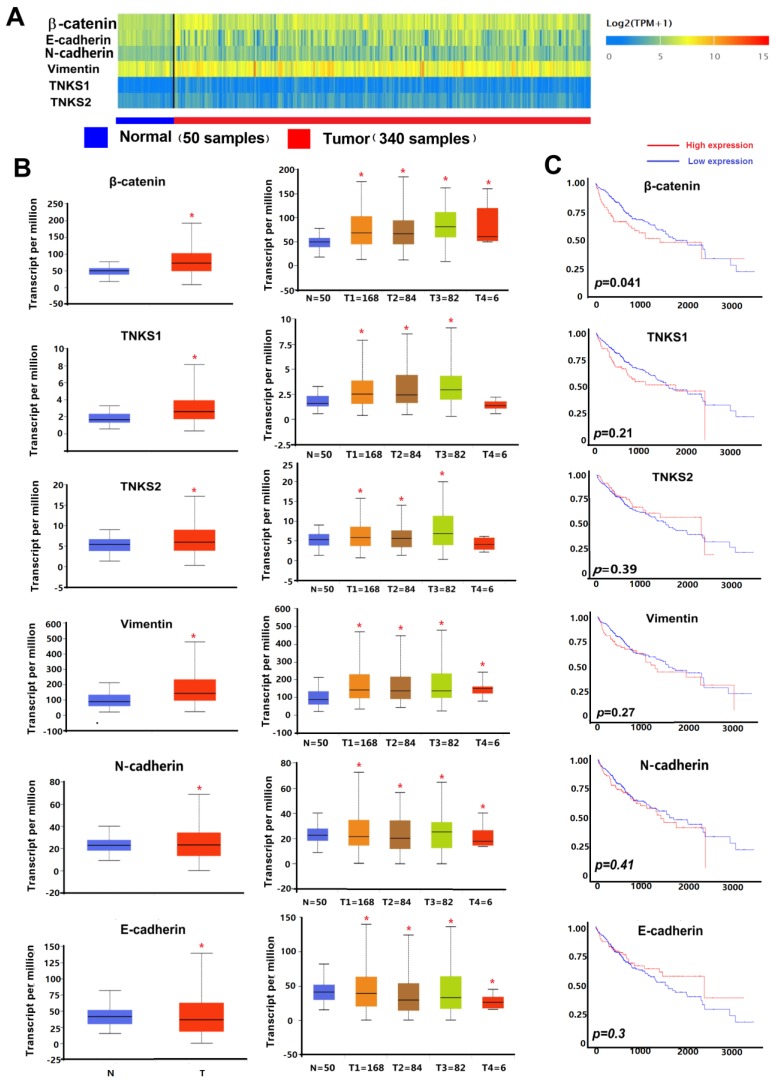
** (A)** Heatmaps showing TNKS/β-catenin pathway-related genes and EMT markers in HCC and adjacent normal tissues. Gene expression levels are represented as log_2_ (transcript count per million [TPM]+1). **(B)** Box-whisker plots showing gene expression in normal samples (N) and HCC sample subgroups of individual stage (T1-T4). **(C)** Kaplan-Meier plots showing the association of high levels (red line) and low levels (blue line) of gene expression with overall survival.

**Figure 2:**
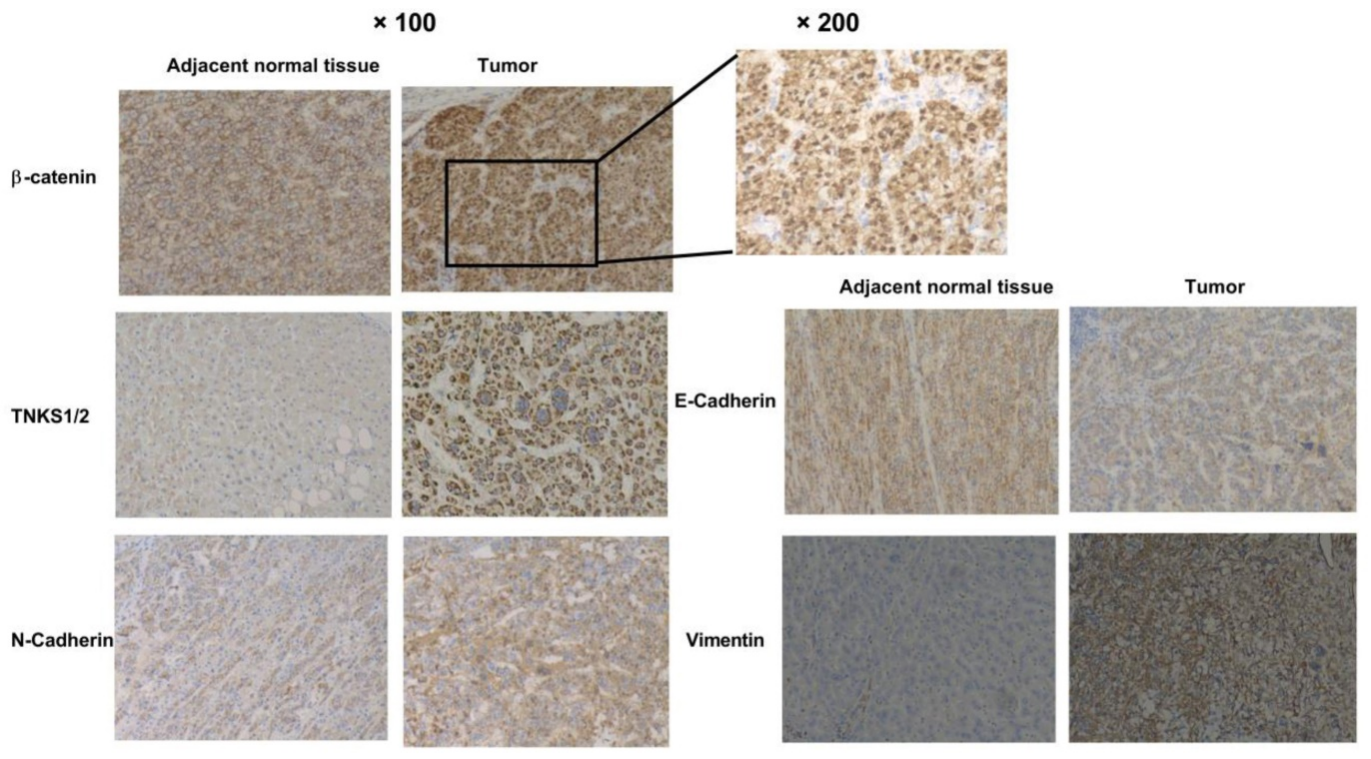
Immunohistochemical analyses of β-catenin, TNKS1/2, E-cadherin, N-cadherin, and vimentin expression in HCC and adjacent normal tissues.

As shown in the heatmap and box-whisker plots of genes in Figure 1, β-catenin, vimentin, TNKS1, TNKS2, and N-cadherin were overexpressed in TCGA HCC samples (n = 340) compared to normal samples (n = 50). Only E-cadherin had a lower expression in the HCC samples (*p*=0.0011). Kaplan-Meier plots showed that only β-catenin expression was associated with HCC patient overall survival (*p*<0.05). As shown in Figure 2, in our IHC study, we also observed the same results, with β-catenin, TNKS1/2, and vimentin and N-cadherin having higher expression levels in 10 HCC tissues compared to the adjacent normal tissues, while the opposite result was observed regarding E-cadherin expression. In addition, the IHC results show that β-catenin was elevated in HCC tissue, especially in the cytoplasm and nucleus. The decreased expression of E-cadherin, and the increased expression of N-cadherin and vimentin, were correlated with increased β-catenin nuclear accumulation in the HCC tissues. These results showed that the TNKS/β-catenin pathway and EMT genes were aberrantly active in HCC, which provides a promising therapeutic target for the abrogation of HCC growth, metastasis, and invasion.

### TNKS inhibition suppressed the proliferation of HCC cell lines

**Figure 3:**
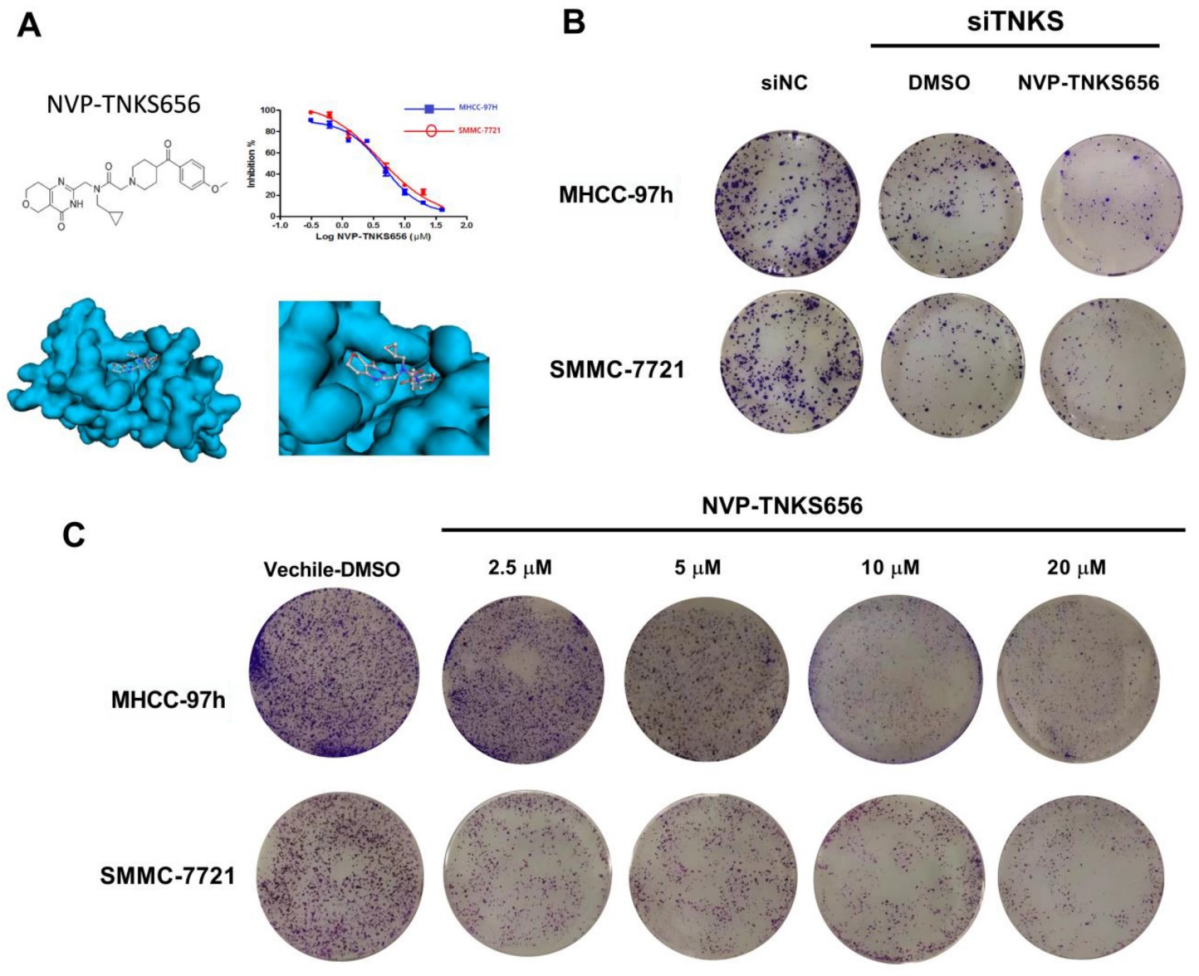
** TNKS inhibition reduced cell growth and colony formation in MHCC-97H and SMMC-7721 cells. (A)** NVP-TNKS656 inhibited HCC cell growth as it binds to the active pocket in the TNKS1 protein. **(B) and (C)** Number of colonies formed by HCC cell lines was reduced after NVP-TNKS656 or siTNKS1/2 treatment.

The TNKS inhibitor NVP-TNKS656 and TNKS knockdown using siRNA were used to investigate the effect of TNKS/β-catenin signaling on the proliferation of HCC cell lines. As shown in Figure 3A, NVP-TNKS656 inhibited the proliferation of SMMC-7721 and MHCC-97h cells in a dose-dependent manner. The molecular docking results revealed that NVP-TNKS656 is a very effective inhibitor that binds precisely to the active pocket in the TNKS1 protein (Figure 3A). The colony formation results also showed that the SMMC-7721 and MHCC-97h cells were inhibited by various concentrations of NVP-TNKS656 (2.5 to 20 μM) in a dose-dependent fashion (Figure 3C). To assess whether TNKS is the target of the β-catenin pathway responsible for cellular proliferation, TNKS1/2 expression was directly inhibited by the siTNKS1/2 mixture. Compared to the siNC group, there were fewer clones in the six-well plates in the TNKS1/2 knockdown group and the 2.5 μM NVP-TNKS656 group. Thus, these results show that TNKS inhibition (by NVP-TNKS656 and siRNA) is responsible for proliferation suppression in HCC cell lines.

### TNKS1/2 knockdown abrogated the metastasis, invasion, and EMT phenotypical features in HCC cell lines

**Figure 4:**
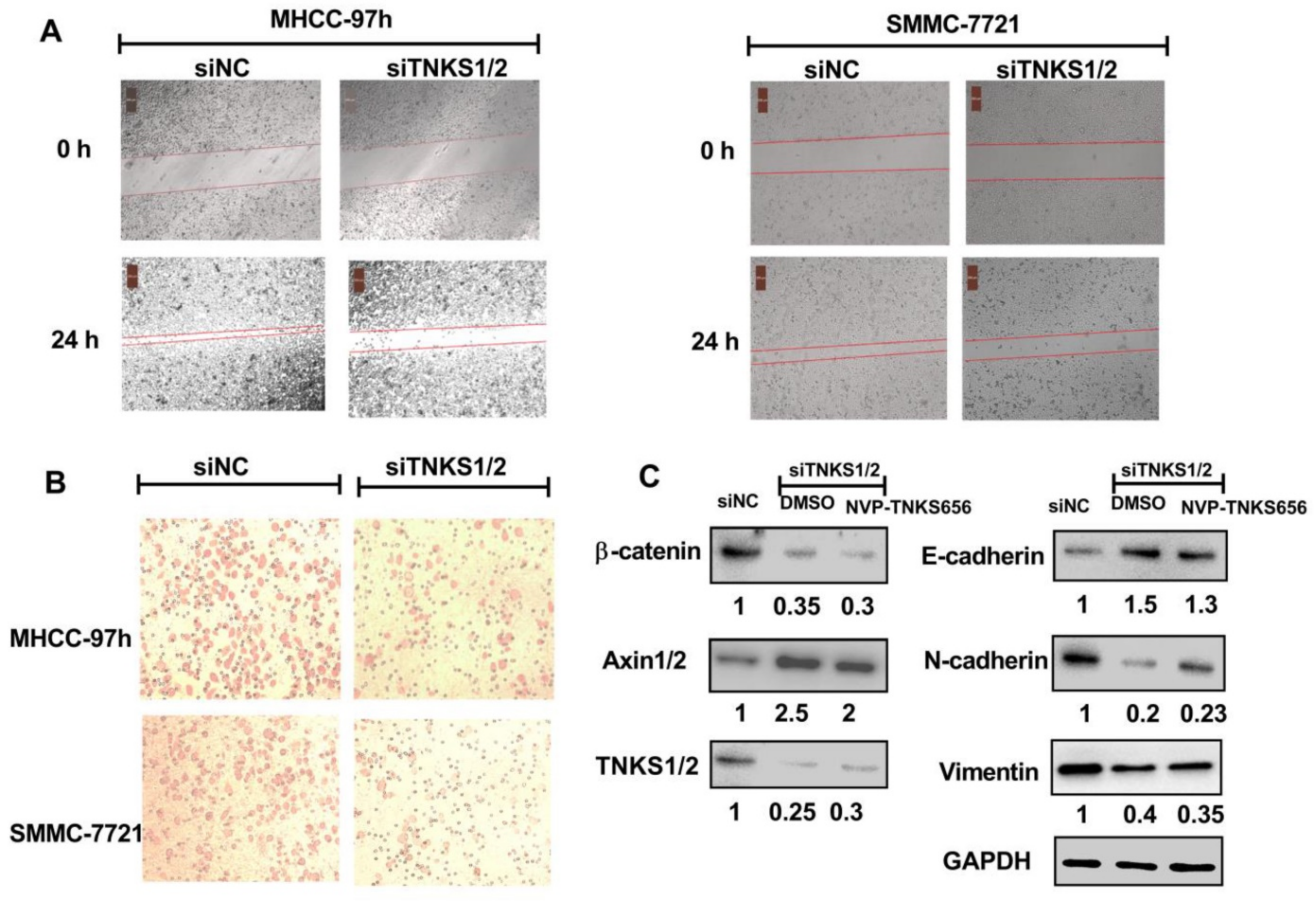
** TNKS1/2 knockdown affected migration, metastasis, and invasion in HCC cells. (A)** Wound-healing assay showing the inhibitory effects of siRNA on TNKS1/2 regarding the migration of HCC cells. **(B)** Cell invasion was measured using Transwell chambers for 36 h. Cells were stained with crystal violet. **(C)** Levels of TNKS/β-catenin pathway-related proteins and EMT markers were detected by western blotting after TNKS1/2 knockdown and 2.5 μM NVP-TNKS656 treatment in MHCC-97h cells.

Given the essential role of TNKS regarding regulating the Wnt/β-catenin signaling pathway in HCC cells, TNKS1/2 knockdown was investigated to assess whether TNKS plays a positive regulatory role in the metastasis, invasion, and EMT characteristics of SMMC-7721 and MHCC-97h cells. As shown in Figure 4, after siTNKS1/2 or siNC transfection for 24 h, the wound-healing assay revealed that TNKS1/2 silencing interrupted SMMC-7721 and MHCC-97h migration, whereas the cells in negative control group (siNC) clearly exhibited migration.

Similar results were also found for the cell invasion assay. The cell invasion assay showed that TNKS1/2 knockdown by siRNA transfection significantly suppressed the cell invasion ability compared to cells transfected with scrambled siRNA (siNC). Further, the western blotting results showed that TNKS/β-catenin signaling was clearly inhibited by TNKS1/2 siRNA transfection in MHCC-97h cells, as indicated by the downregulation of β-catenin and TNKS1/2, as well as the upregulation of AXIN1/2. The EMT genes, N-cadherin and vimentin, were also decreased after TNKS1/2 siRNA transfection or combination with 2.5 μM NVP-TNKS656, along with elevated levels of E-cadherin. These findings indicate that TNKS1/2 knockdown was responsible for β-catenin downregulation and the further abrogation of metastasis, invasion, and EMT in HCC cell lines.

### NVP-TNKS656 inhibited metastasis, invasion, and EMT in HCC cell lines

**Figure 5:**
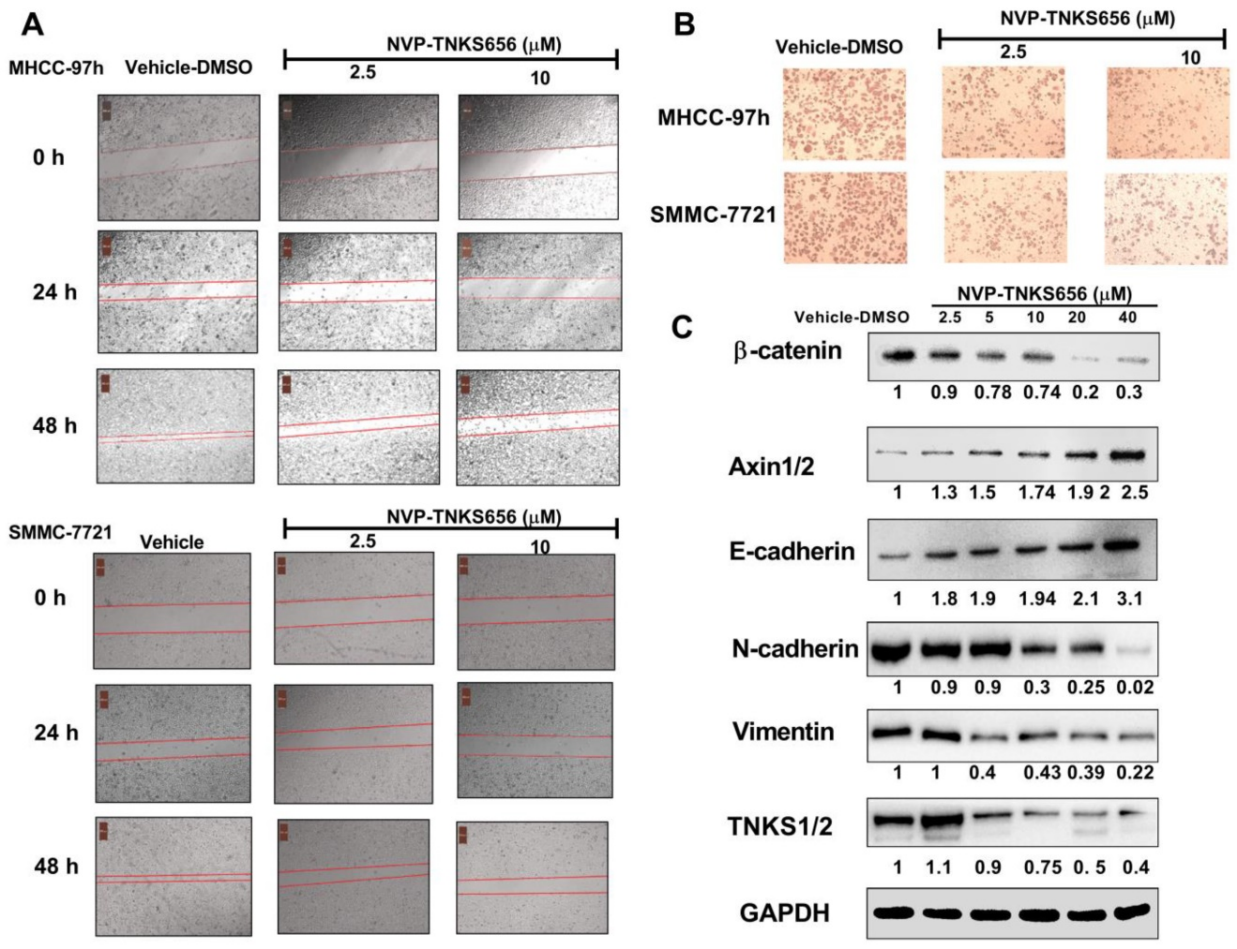
** NVP-TNKS656 affected migration, metastasis, and invasion in HCC cells. (A)** Wound-healing assay showing the inhibitory effects of NVP-TNKS656 on the migration of HCC cells. **(B)** Cell invasion was assessed using Transwell chambers for 36 h. Cells were stained with crystal violet. **(C)** Levels of TNKS/β-catenin pathway-related proteins and EMT markers were detected by western blotting after exposure to various concentrations of NVP-TNKS656 and vehicle control (DMSO) in MHCC-97h cells.

To further explore the use of TNKS as a therapeutic target for the inhibition of metastasis, invasion, and EMT in HCC cell lines, NVP-TNKS656, a TNKS inhibitor, was used to treat HCC cell lines. As shown in Figure 5, after exposure to NVP-TNKS656 (2.5 or 10 μM), the migration abilities of MHCC-97h and SMMC-7721 cells were inhibited in the wound-healing assay, with the same results as for invasion inhibition in the Transwell invasion assay. The levels of TNKS/β-catenin pathway-related proteins and EMT markers were further detected by western blotting after exposure to various concentrations of NVP-TNKS656 (2.5 to 40 μM) and vehicle control (0.1% DMSO) in MHCC-97h. In contrast to the vehicle control group, β-catenin, N-cadherin, vimentin and TNKS1/2 were reduced by NVP-TNKS656 in a dose-dependent manner, whereas AXIN1/2 and E-cadherin were upregulated by NVP-TNKS656 in MHCC-97h.

## Discussion

The present study showed that the aberrant expression of TNKS/β-catenin pathway-related genes and EMT markers in HCC patients could act as therapeutic targets. TNKS inhibition in HCC cell lines (by siRNA or the inhibitor NVP-TNKS656) further indicated that the TNKS/β-catenin signaling pathway is an anti-proliferation and anti-metastatic target in HCC cell lines.

Numerous studies have shown that the canonical Wnt/β-catenin signaling pathway plays a vital role in HCC 2, 26-28. The high expression of β-catenin and the activation of the nuclear β-catenin (CTNNB1)/TCF/LEF transcriptional complex are recognized as early events in hepatocarcinogenesis. Moreover, in addition to β-catenin (CTNNB1) gene mutation, other mutations of the destruction complex members, such as AXIN1, AXIN2, APC, and GSK3β 6, 29, are also involved in the aberrant regulation of the Wnt/β-catenin signaling pathway in HCC. Thus, previous findings indicate that the Wnt/β-catenin signaling pathway and related complexes are potentially effective therapeutic targets. In the present study, the IHC results show that β-catenin was elevated in HCC tissue, especially in the cytoplasm and nucleus. Moreover, β-catenin was significantly associated with overall survival. These results imply that the TNKS/β-catenin pathway is aberrantly active in HCC, which provides us a promising therapeutic target for the abrogation of HCC growth, metastasis, and invasion.

EMT is an essential step in HCC progression that potentiates malignant tumor cell invasion and metastasis, thereby leading to death in HCC patients 30. Studies have shown that the upregulation of Wnt/β-catenin signaling promoted hepatocarcinogenesis and enhanced cell invasion 31, whereas the abrogation of Wnt/β-catenin signaling blocked the migration and invasion of HCC cell lines 32. The IHC results in the present study indicate that higher expression levels of EMT markers (including N-cadherin and vimentin) were found in HCC tissues compared to adjacent normal tissues, but lower E-cadherin levels were found, which is consistent with previous reports 33. Research has shown that a disruption of E-cadherin/β-catenin complexes at cell boundaries is characteristic of hepatocellular EMT, and this disruption occurs in experimental HCC in mice 33, 34. Reduced E-cadherin expression has been found to be accompanied by (partial) nuclear translocation of β-catenin, and it was significantly correlated with intrahepatic metastasis and poor patient survival 33, 35. Our present results also indicate that the increased expression of N-cadherin and vimentin, and the decreased expression of E-cadherin, were correlated with increased β-catenin nuclear accumulation in the HCC tissues. These results suggest that the disruption of TNKS/β-catenin pathway in HCC probably remodels EMT marker expression.

TNKS1 and TNKS2, positive regulators of β-catenin activation, were reported to be overexpressed in HCC samples 21, which was also shown by our IHC experiments and UALCAN web-portal analysis. siRNA-mediated knockdown of TNKS1 and TNKS2 reduced MHCC-97h and SMMC-7721 cell proliferation and colony formation ability via the suppression of endogenous TNKS1 and TNKS2, along with decreasing β-catenin levels. A previous study using HepG2 and Huh7 cells reported similar results 21. Furthermore, after RNA interference using siRNA in MHCC-97h and SMMC-7721 cells, the wound-healing and Transwell invasion assays revealed that these HCC cell lines exhibited significantly decreased migration and invasion abilities. Concurrently, EMT marker expression was restored, including increased E-cadherin expression and decreased vimentin and N-cadherin levels. The EMT phenotypic features in the HCC cell lines were probably changed via TNKS1/2 knockdown and subsequent β-catenin pathway abrogation.

Regarding to the roles of TNKS1 and TNKS2 as positive regulators of β-catenin pathway activation, many TNKS inhibitors have been discovered and characterized 36. NVP-TNKS656 is an orally active antagonist of Wnt pathway activity that was identified in the MMTV-Wnt1 mouse xenograft model 23. NVP-TNKS656 promoted apoptosis in phosphoinositide 3-kinase (PI3K) or AKT inhibitor-resistant CRC cells with a high nuclear β-catenin content 37. Based on the previous findings, NVP-TNKS656 was used to inhibit the β-catenin pathway by binding to the dual active pockets in the TNKS protein 23. With increasing concentrations of NVP-TNKS656, MHCC-97h and SMMC-7721 cell proliferation and clone formation were both inhibited in a dose-dependent fashion. Moreover, EMT features in MHCC-97h and SMMC-7721 cells were also changed by NVP-TNKS656. The results of the wound-healing and invasion assays show that the migration and invasion abilities of the MHCC-97h and SMMC-7721 cells were dramatically decreased after exposure to NVP-TNKS656. The western blotting results indicate that NVP-TNKS656 probably influenced EMT marker expression via the stabilization of AXIN levels, which led to reduced β-catenin levels, thereby restoring the EMT characteristics and decreasing the invasion and migration abilities.

Taken together, our results suggest that TNKS inhibition decreased the invasion and metastasis capacity in HCC cell lines by affecting β-catenin-mediated EMT. The related signaling pathway may offer potential targets for HCC cancer therapy. However, β‑catenin inhibitors may also be applicable for the prevention of organ fibrosis, second‑line HCC prevention, and treating β‑catenin‑driven cancer. Multilayered prevention and treatment strategies for β‑catenin‑related human cancers are necessary for the implementation of personalized precision medicine.

## Abbreviations

ANK: ankyrin
APC: adenomatous polyposis coli
CSNK1A1: casein kinase 1 alpha 1
CTNNB1: β-catenin
DMEM: dulbecco's modified eagle medium
EMT: epithelial-mesenchymal transition
FBS: fetal bovine serum
GSK3β: glycogen synthase kinase 3 beta
HCC: Hepatocellular carcinoma
IHC: immunohistochemistry
PBS: phosphate-buffered saline
PARPs: poly ADP ribosyl polymerases
SDS-PAGE: sodium dodecyl sulfate polyacrylamide gel electrophoresis
SAM: sterile alpha molecule
TCGA: The Cancer Genome Atlas
TNKS: tankyrase
